# A Brief Review of the Effects of Vitamin D on Multiple Sclerosis

**DOI:** 10.3389/fimmu.2020.00781

**Published:** 2020-05-06

**Authors:** Andrei Miclea, Maud Bagnoud, Andrew Chan, Robert Hoepner

**Affiliations:** Department of Neurology, University Hospital Bern and University of Bern, Bern, Switzerland

**Keywords:** metabolism, guidelines, nervous system, MS risk, disease activity, innate adaptive immune system, calcitriol, cholecalciferol

## Abstract

Multiple sclerosis (MS) is characterized as an autoimmune disease affecting the central nervous system. It is one of the most common neurological disorders in young adults. Over the past decades, increasing evidence suggested that hypovitaminosis D is a contributing factor to the risk of developing MS. From different risk factors contributing to the development of MS, vitamin D status is of particular interest since it is not only a modifiable risk factor but is also associated with MS disease activity. MS patients with lower serum vitamin D concentrations were shown to have higher disease activity. However, this finding does not demonstrate causality. In this regard, prospective vitamin D supplementation studies missed statistical significance in its primary endpoints but showed promising results in secondary outcome measures or *post hoc* analyses. An explanation for missed primary endpoints may be underpowered trials. Besides vitamin D supplementation as a potential add-on to long-term immunotherapeutic treatment, a recent laboratory study of our group pointed toward a beneficial effect of vitamin D to improve the efficacy of glucocorticoids in relapse therapy. In the following article, we will briefly review the effects of vitamin D on MS by outlining its effects on the immune and nervous system and by reviewing the association between vitamin D and MS risk as well as MS disease activity. We will also review the effects of vitamin D supplementation on MS risk and MS disease activity.

## Introduction

**FIGURE 1:**
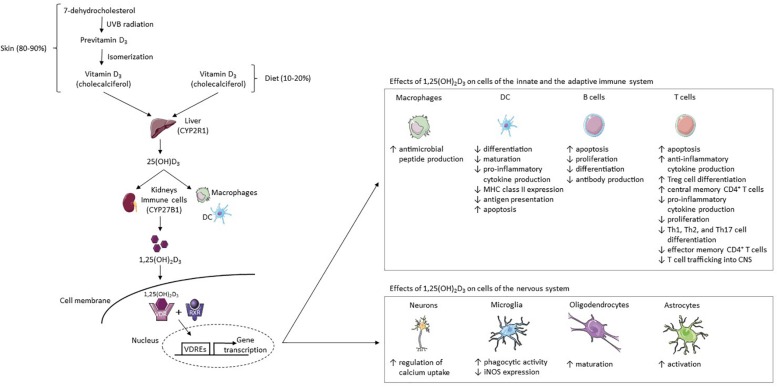
Vitamin D_3_ metabolism and its effects on cells of the immune and nervous system. 80–90% of the body’s vitamin D supply is produced by the skin’s exposure to UVB radiation and 10–20% is acquired through diet (7). Fatty fish contain high amounts of vitamin D_3_ (cholecalciferol) (8). In the skin, the vitamin D_3_ precursor 7-dehydrocholesterol converts to previtamin D_3_ after UVB exposure (10, 11). Previtamin D_3_ then isomerizes to cholecalciferol (10, 11). This physiologically inactive form of vitamin D_3_ is hydroxylated in the liver to 25(OH)D_3_ by CYP2R1 (13). It is then hydroxylated by the enzyme CYP27B1 in the kidneys or at inflammatory sites by immune cells such as DCs and macrophages, resulting in the fully-active metabolite 1,25(OH)_2_D_3_ (12, 14). In target cells, 1,25(OH)_2_D_3_ binds to the VDR, thereafter forming a complex with the RXR-γ (15, 16). The 1,25(OH)_2_D_3_-VDR-RXR-γ complex binds certain DNA sequences (VDREs), thereby modulating gene transcription (15–17). 1,25(OH)_2_D_3_ increases the production of antimicrobial peptides from macrophages, while in the DC line it inhibits (I) monocyte differentiation into DCs, (II) DC maturation, (III) production of pro-inflammatory cytokine IL-12, (IV) MHC class II expression, (V) and antigen presentation (46, 47, 52–55). DCs are induced to undergo apoptosis (55). 1,25(OH)_2_D_3_ increases (I) T cell apoptosis, (II) anti-inflammatory cytokine production, (III) Treg cell differentiation, and (IV) the proportion of central memory CD4 + T cells (55–58, 67, 77, 78). In addition, it decreases (I) pro-inflammatory cytokine production, (II) T cell proliferation, (III) Th1, Th2, and Th17 cell differentiation, (IV) the proportion of effector memory CD4 + T cells, and (V) T cell trafficking into the CNS (55–57, 67, 78, 88). In B cells, 1,25(OH)_2_D_3_ increases apoptosis and reduces proliferation, differentiation and antibody production (44, 59–61). 1,25(OH)_2_D_3_ increases regulation of calcium uptake in neurons and phagocytic activity in microglia but reduces iNOS expression in microglia (97, 98). Lastly, 1,25(OH)_2_D_3_ stimulates oligodendrocyte maturation and astrocyte activation (99). Abbreviations: CYP2R1, vitamin D_3_ 25-hydroxylase; CYP27B1, 25(OH)D_3_-1α-hydroxylase; DC, dendritic cell; iNOS, inducible nitric acid synthase; MHC, major histocompatibility complex; RXR, retinoid x receptor; UVB, ultraviolet B; VDR, vitamin D receptor; VDREs, vitamin D response elements; 1,25(OH)_2_D_3_, 1,25-dihydroxyvitamin D_3_; 25(OH)D_3_, 25-hydroxyvitamin D_3_. This figure was created using Servier Medical Art templates licensed under a Creative Commons License (https://creativecommons.org/licenses/by/3.0/).

The exact pathophysiological mechanisms leading to the development of multiple sclerosis (MS) are not fully understood (1, 2). Nonetheless, certain genetic and environmental factors influencing not only MS risk but also MS disease activity have been identified (1, 3–6). One of the identified factors is vitamin D status (1, 4). In this article, we will briefly review the effects of vitamin D on MS. First, we will review the metabolism (summarized in Figure 1), the biological and safety features, and the intake guidelines of vitamin D. Second, we will outline the effects of vitamin D on cells of the innate and adaptive immune system and cells of the nervous system (summarized in Figure 1). Third, the association of vitamin D and MS risk as well as MS disease activity will be laid out (summarized in Table 1). Lastly, the evidence of the effects of vitamin D supplementation on MS risk, MS disease activity, and as a potential add-on for relapse therapy will be outlined (summarized in Table 1).

**TABLE 1:** Vitamin D and its association with MS.

**Vitamin D and its association with MS risk**
MS susceptibility gene HLA-DRB1*1501 regulated by a vitamin D dependent promoter (54)
Higher risk of MS at higher latitudes (100, 101)
Higher risk of MS in people with a genetic predisposition to vitamin D deficiency (103–105)
Higher risk of MS in offspring of mothers with vitamin D deficiency during early pregnancy (106)
Higher risk of MS in neonates with low serum 25(OH)D concentration (107)
Higher risk of MS/CIS in adults with lower serum 25(OH)D concentration (6, 108)
Gradual decrease in serum 25(OH)D concentration in the 24-month period prior to onset of CIS (108)
Vitamin D supplementation associated with a 40% lower MS risk (123)
**Vitamin D status and its association with MS disease activity**
Serum 25(OH)D concentration inversely correlated with relapse risk (117, 118, 121)
Serum 25(OH)D concentration inversely correlated with CNS lesions (120)
Serum 25(OH)D concentration inversely correlated with disability progression (121)
BENEFIT: 50 nmol/L higher serum 25(OH)D concentration subsequently associated with 57% lower relapse rate, 57% lower rate of new active lesions, and lower disability progression (116)
BEYOND: 50 nmol/L higher serum 25(OH)D concentration subsequently associated with 31% lower risk of new lesions, but no significant differences in relapse risk and disability progression (119)
**Vitamin D supplementation and MS disease activity**
SOLAR: Number of new gadolinium-enhancing or new/enlarging T2 lesions significantly reduced through cholecalciferol, yet no significant difference in ARR and disability progression (36)
CHOLINE: ITT population: No significant ARR reduction through cholecalciferol, yet in study completers significant reduction in ARR, new T1 lesions, and disability progression (125)
No significant improvement in depression scores through supplementation with cholecalciferol (131)
Significant reduction of fatigue scores through supplementation with alfacalcidol but not with cholecalciferol (131, 132)
**Vitamin D supplementation and MS relapse therapy**
1,25(OH)_2_D_3_ increases glucocorticoid induced effects both *in vitro* and *in vivo* (138)

*ARR, annualized relapse rate; CIS, clinically isolated syndrome; CNS, central nervous system; ITT, intention to treat; MS, multiple sclerosis; 1,25(OH)_2_D_3_, 1,25-dihydroxyvitamin D_3_; 25(OH)D, 25-hydroxyvitamin D.*

## Vitamin D and Its Metabolism, Its Biological Features, Its Intake Guidelines, and Its Safety Considerations

In humans, between 80 and 90% of the body’s vitamin D supply is produced by the skin’s exposure to ultraviolet B (UVB) radiation and only 10–20% is acquired through diet (7). Fatty fish, e.g., salmon, sardines, and tuna, contain relatively high amounts of vitamin D_3_ (cholecalciferol), while plants provide vitamin D_2_ (ergocalciferol) (8, 9). In the skin, the vitamin D_3_ precursor 7-dehydrocholesterol converts to previtamin D_3_ after UVB exposure (10, 11). Previtamin D_3_ then isomerizes to cholecalciferol (10, 11). Ergocalciferol and cholecalciferol are physiologically inactive forms of vitamin D, which are hydroxylated in the liver to 25-hydroxyvitamin D_2_ [25(OH)D_2_] and 25-hydroxyvitamin D_3_ [25(OH)D_3_], respectively (12, 13). Because the human body’s vitamin D supply is largely provided by the skin’s production of cholecalciferol, the major circulatory form of 25(OH)D is 25(OH)D_3_ being produced in the liver by the key enzyme vitamin D_3_ 25-hydroxylase (CYP2R1) (7, 13). 25(OH)D_3_ is then hydroxylated by the enzyme 25(OH)D_3_-1α-hydroxylase (CYP27B1) in the kidneys or at inflammatory sites by immune cells such as dendritic cells and macrophages, resulting in the fully-active metabolite 1,25-dihydroxyvitamin D_3_ [1,25(OH)_2_D_3_] (calcitriol) (12, 14). In target cells, 1,25(OH)_2_D_3_ binds to the vitamin D receptor (VDR), thereafter forming a complex with the retinoid x receptor γ (RXR-γ) (15, 16). The 1,25(OH)_2_D_3_-VDR-RXR-γ complex then binds certain DNA sequences called vitamin D response elements (15–17). In consequence, transcription rates of genes involved in a whole array of different functions of the human body are modified, including regulation of the immune system as well as cellular proliferation and differentiation (18). The VDR genotype is associated with different autoimmune disorders such as type 1 diabetes mellitus, Grave’s disease, Addison’s disease, and rheumatoid arthritis (19–23). 1,25(OH)_2_D_3_ is inactivated via a third hydroxylation by the enzyme CYP24A1 to 1,24,25(OH)_3_D_3_, which is present in target cells expressing the VDR (12, 24). The VDR is present in many human tissues explaining the various effects of 1,25(OH)_2_D_3_ on the human body, yet it is predominantly expressed in the intestines, the pancreas, the kidneys, and osteoblasts (25). In addition to 1,25(OH)_2_D_3_, also 25(OH)D_3_ binds to the VDR (26). However, 1,25(OH)_2_D_3_ has a higher affinity to the VDR and is, therefore, the more active metabolite (26).

1,25(OH)_2_D has a relatively short half-life (4 h) compared to 25(OH)D (2–3 weeks) (27, 28). Due to its relatively stable properties, serum 25(OH)D concentration serves as the common surrogate of the body’s vitamin D status (29). In the general population, the Endocrine Society considers serum 25(OH)D concentrations <20 ng/mL (50 nmol/L) to signal vitamin D deficiency, concentrations between ≥20 ng/mL and <30 ng/mL (75 nmol/L) to be insufficient, ≥30 ng/mL to be sufficient, and concentrations ≤100 ng/mL (250 nmol/L) to be safe (conversion factor: 1 ng/mL = 2.5 nmol/L) (30–32). However, recommendations on optimal serum 25(OH)D concentrations differ between medical societies (31, 33, 34). Since 40% of adults have 25(OH)D concentrations of <20 ng/mL (50 nmol/L), it is evident that the “western” lifestyle and diet are not sufficient for an adequate vitamin D supply (35). In vitamin D deficient adults, the Endocrine Society suggests to supplement with 6,000 IU/d for 8 weeks and afterward to maintain serum 25(OH)D concentrations >30 ng/mL by prescribing 1,500 – 2,000 IU/d of cholecalciferol or ergocalciferol, which is the required maintenance dose of desirable 25(OH)D concentrations (>30 ng/mL) (31). The upper tolerable intake limit is set at 10,000 IU/d (31). Nonetheless, in a randomized clinical trial (*N* = 229) comparing placebo and 14,007 IU/d of cholecalciferol over 48 weeks in MS patients receiving beta interferons, the occurrence of adverse events was similar in the cholecalciferol plus interferons group and in the placebo plus interferons group (36). However, since patient numbers are low, the occurrence of side effects caused by such a vitamin D dose cannot be ruled out (36). Vitamin D intoxication might become clinically relevant in persons using very high doses (mostly >50,000 IU/d), resulting in serum 25(OH)D concentrations ≥150 ng/mL (375 nmol/L) (37). These doses and serum concentrations can lead to hypercalcemia, hypercalciuria, and hyperphosphatemia, which can manifest as nausea and emesis, muscle weakness, polyuria, calcification of the kidneys, and in extreme cases kidney failure (37). Therefore, ultra-high-dose vitamin D regimens such as the “Coimbra-protocol” in MS with suggested doses of up to 400,000 IU/d pose a considerable safety hazard for patients (38). In this regard, Häusler et al. demonstrated in a rodent animal model of MS, experimental autoimmune encephalitis (EAE), that prolonged high-dose vitamin D supplementation can lead to disease exacerbation if serum 25(OH)D concentrations >80 ng/mL (200 nmol/L) were reached (39). However, disease exacerbation seemed to be mediated primarily by vitamin D induced hypercalcemia rather than 1,25(OH)_2_D_3_ itself because hypercalcemia induced the activation of T cells leading to the migration of activated myeloid, Th1, and Th17 cells into the central nervous system (CNS) (39). Similar 25(OH)D concentrations in humans (>64 ng/mL, or 160 nmol/L) lead only in approximately 10% of patients to hypercalcemia (40). Therefore, the translational significance of autoimmune disease exacerbation through high-dose vitamin D supplementation remains unclear.

## The Effects of Vitamin D on the Innate and the Adaptive Immune System

Not only MS but also several other autoimmune disorders are associated with vitamin D deficiency (1, 41–43). Accordingly, studies performed *in vitro* and *in vivo* have shown that 1,25(OH)_2_D_3_ has anti-inflammatory effects by suppressing the innate as well as the adaptive immune system (44).

Regarding the innate immune system, after phagocytosis of microbes through macrophages, Toll-like receptors are activated, resulting in an up-regulation of VDR and CYP27B1 expression in macrophages and monocytes (45). In macrophages, 1,25(OH)_2_D_3_ then activates cathelicidins, which are antimicrobial peptides (46, 47). Another anti-inflammatory mechanism of action of 1,25(OH)_2_D_3_ is exerted through its various effects on glucocorticoids, including an increased stimulation of monocytes by glucocorticoids to produce mitogen-activated kinase phosphatase 1, which reduces the pro-inflammatory activity of mitogen-activated protein kinases (48, 49).

Addressing immune cells, which are part of the innate and the adaptive immune system, 1,25(OH)_2_D_3_ increases the differentiation of hematopoietic stem cells into natural killer cells and inhibits the function of the dendritic cell line (50, 51). Regarding the dendritic cell line, 1,25(OH)_2_D_3_ inhibits (I) the differentiation of monocytes into dendritic cells, (II) the maturation of dendritic cells, (III) the production of pro-inflammatory cytokine IL-12, (IV) the expression of the major histocompatibility complex class II, and (V) the presentation of antigens (52–55). Furthermore, dendritic cells are induced to undergo apoptosis (55).

Mediated by its effects on dendritic cells, 1,25(OH)_2_D_3_’s influence on the adaptive immune system has been attributed to its various effects on T cells, including the altered production of cytokines and selective induction of T cells into apoptosis (55–58). Thus far, the effect of 1,25(OH)_2_D_3_ on B cells remains inconclusive as some of the *in vitro* experiments could not be replicated *in vivo* (59). *In vitro*, B cell proliferation and B cell differentiation into plasma cells are inhibited and B cell apoptosis is induced, resulting in the reduced production of antibodies (44, 60, 61). Accordingly, higher serum immunoglobulin G concentrations were associated with lower serum 25(OH)D concentrations in patients with cystic fibrosis (62). In a study of 40 MS patients, however, immunoglobulin G (IgG) concentrations and 25(OH)D concentrations did not significantly correlate in CSF or serum (63). This could be another indicator that the response of MS patients to vitamin D is reduced as shown by Bhargava et al. (64).

Furthermore, in the presence of 1,25(OH)_2_D_3_, the stimulation of T cells by B cells is impaired *in vitro* (65). In reverse, 1,25(OH)_2_D_3_ is produced by T cells as these express CYP27B1 (66). Most experimental studies found that 1,25(OH)_2_D_3_ acts on CD4 + T cells (Th1, Th2, Th17, Treg cells) by inhibiting their proliferation and their secretion of pro-inflammatory cytokines (IL-2, IL-17, IFN-γ) and by stimulating their secretion of anti-inflammatory cytokines (IL-4, IL-10) (57, 67–76). In consequence, the cytokine profile is skewed from a Th1 (decrease of IFN-γ) to a Th2 mediated profile (increase of IL-4) (75, 76). Furthermore, the differentiation of Th1, Th2, and Th17 cells is inhibited and Treg cell differentiation is induced by 1,25(OH)_2_D_3_ (67, 77). A double-blind prospective study in MS patients confirmed the finding that vitamin D supplementation reduces IL-17 production by CD4 + T cells (78). Additionally, it demonstrated an increased proportion of central memory CD4 + T cells and naive CD4 + T cells but a decrease in the proportion of effector memory CD4 + T cells (78).

## The Effects of Vitamin D on the Nervous System and on EAE

Neural cells express the VDR, 1,25(OH)_2_D_3_ is synthesized by neurons and microglia, and cerebrospinal fluid (CSF) 25(OH)D concentration significantly correlates with its concentration in the plasma (79–83). In contrast to a study performed by Balabanova et al. in the 1980s, a more recent (2009) study by Holmøy et al. found a substantially lower ratio of CSF to serum 25(OH)D concentration (0.57:1 vs. 0.006:1) (83). Nonetheless, both studies showed a correlation between 25(OH)D concentrations in CSF and serum (82, 83). 1,25(OH)_2_D_3_ modulates neurotrophic factors and regulates the influx of calcium into neurons through the interaction with L-type calcium channels (84–87). The ability of 1,25(OH)_2_D_3_ to suppress the progression of EAE is attributed to its modulation of T cell trafficking into the CNS, its inhibition of Th1 cells, and its stimulation of IL-10 production (79, 88–95). 1,25(OH)_2_D_3_ induces Indoleamine 2,3-dioxygenase-positive (IDO^+^) tolerogenic dendrocytes and Treg in the periphery and concomitantly reduces the number of autoreactive T cells in the CNS, thereby reducing the severity of guinea pig MBP_73–86_ EAE in lewis rats (96). Demyelination is reduced via 1,25(OH)_2_D_3_’s activation of microglia resulting in the clearance of myelin debris, phagocytosis of pathological proteins such as amyloid-β peptides, and the reduced expression of inducible nitric acid synthase, which is a pro-inflammatory enzyme (97, 98). Lastly, 1,25(OH)_2_D_3_ might induce remyelination by stimulating the maturation of oligodendrocytes and the activation of astrocytes in female C57Bl/6 mice demyelinated with cuprizone (99).

## Vitamin D Status and Its Association With MS Risk

An important clinical association between MS and vitamin D is that populations located farther from the equator and, therefore, receiving less exposure to UVB radiation face more frequently vitamin D deficiency and simultaneously a higher risk of MS (100, 101). In observational studies, the distinction of the effects of UVB radiation and the effects of vitamin D on MS risk is only insufficiently adjusted for as also UVB radiation is able to suppress the development of MOG_35–55_ EAE (102). However, since Ramagopalan et al. found the MS susceptibility gene HLA-DRB1^∗^1501 to be regulated by a vitamin D dependent promotor, a clinically relevant UVB independent effect of vitamin D on MS risk appears feasible (54). To attempt to answer the question whether low 25(OH)D causes MS or MS causes low 25(OH)D, different research groups performed Mendelian randomization studies (103–105). Concordantly, these studies found a higher likelihood of developing MS if patients’ genes predetermined them to have lower 25(OH)D concentrations leading to the conclusion that 25(OH)D concentrations indeed influence MS risk (103–105). A Scandinavian study reported an almost two-fold risk of developing MS in the offspring of mothers with 25(OH)D concentrations <12.02 ng/mL (30.05 nmol/L) during early pregnancy (106). Accordingly, neonates with serum 25(OH)D concentrations in the bottom quintile (<8.28 ng/mL, or 20.7 nmol/L) had the highest likelihood of developing MS and neonates in the upper quintile (≥19.56 ng/mL, or 48.9 nmol/L) the lowest likelihood (107). In the 24-month period prior to the development of clinically isolated syndrome (CIS), our research group demonstrated that patients not only showed significantly lower 25(OH)D concentrations in comparison to healthy controls but that they also showed a gradual decrease in 25(OH)D concentrations as the incident of the first clinical manifestation of MS approached (108). 1,25(OH)_2_D_3_ and the vitamin D receptor were also shown to interact with Epstein-Barr virus (EBV) nuclear antigens (EBNA), which are thought of as key contributors to MS pathogenesis (3, 109–111). It is hypothesized that hypovitaminosis D increases the autoimmune effects of EBV infection, thereby increasing the risk of developing MS because of the following reasons (111). First, in young MS patients, antibody reactivity against EBNA-1 increases with lower 25(OH)D levels (109). Second, anti-EBNA 1 protein and fragment antibody concentrations decrease after vitamin D supplementation in comparison to placebo (112, 113). Third, EBNA 2 and the VDR have common DNA binding sites associated with MS (114). Lastly, the activation of VDR target genes is blocked by EBNA 3 binding to the VDR (115).

## Vitamin D Status and Its Association With Disease Activity

Not only is MS risk associated with low 25(OH)D concentration but also certain parameters of MS disease activity (116–121). In a *post hoc* analysis of patients with CIS included in a randomized placebo-controlled clinical trial originally designed to investigate the effects of early versus delayed treatment with interferon beta-1b (BENEFIT), patients with a 20 ng/mL (50 nmol/L) higher serum 25(OH)D concentration had subsequently a 57% lower relapse rate and a 57% lower rate of new active lesions (116). Conflictingly, in an analysis of MS patients, included in a randomized placebo-controlled clinical trial to investigate two different doses of interferon beta-1b (BEYOND), serum 25(OH)D concentrations were not significantly associated with subsequent relapse rates (119). Regarding CNS lesions, however, the analysis of BEYOND patients showed that a 20 ng/mL (50 nmol/L) higher serum 25(OH)D concentration was associated with a 31% lower risk of new lesions (119). Next to relapse rate, conflicting evidence also exists regarding disability progression as the *post hoc* analysis of BENEFIT reported an inverse correlation between 25(OH)D concentration and subsequent disability progression, yet the analysis of BEYOND patients could not confirm this finding (116, 119). In populations with MS, initial findings of an inverse correlation between serum 25(OH)D concentration and depression as well as fatigue were non-significant after adjusting for UVB exposure as a confounder (122). The authors concluded that sunlight exposure was more robustly associated with depression scores and fatigue than 25(OH)D concentrations (122).

## Vitamin D Supplementation and Its Association With MS Risk and Disease Activity

In a large observational study including >187,000 women, 173 of whom developed MS, Munger et al. demonstrated that women using vitamin D supplements had a 40% reduced risk of developing MS in the follow-up period (1980–2001) compared to non-users (123). However, because of the observational character, it is possible that relevant confounding factors existed even though the authors adjusted for known MS risk factors such as age, smoking, and latitude of residence at birth (123).

The largest study investigating the effects of vitamin D supplementation on MS disease activity is the SOLAR trial, which was a randomized, double-blind, placebo-controlled trial investigating supplementation with 14,007 IU/d of cholecalciferol for 48 weeks in 229 relapsing-remitting MS patients treated with interferon beta-1a (36). The number of new gadolinium-enhancing or new/enlarging T2 lesions was significantly reduced by 32% in patients supplemented with cholecalciferol in comparison to supplementation with placebo (*p* = 0.0045), yet no significant results were reported regarding annualized relapse rate (ARR) and disability progression (36). Nonetheless, a non-significant trend toward a lower ARR in patients treated with cholecalciferol became evident (0.28 vs. 0.41, *p* = 0.17) (36). Significant results in radiological but non-significant results in clinical disease parameters could be due to higher incidence rates of new/enlarging lesions than relapses or sustained disability progression (124). However, the main reason for missed significance may have been the violation of the power calculation (36). Due to difficulties in patient recruitment, the study duration was shortened from 96 weeks to 48 weeks and the randomized total patient number was reduced by a third from 348 to 232 (36). Furthermore, the primary endpoint was changed from the mean number of active T2 lesions and the patient proportion with no relapses to the patient proportion with no evidence of disease activity (NEDA) 3 defined as no relapses, no disability progression, and no new gadolinium-enhancing or new/enlarging T2 lesions, which was nonetheless missed (36). Camu et al. also investigated in a randomized clinical trial the effects of vitamin D supplementation in patients receiving interferons (125). In comparison to the SOLAR trial, this study was performed over a longer duration (96 weeks) but with fewer patients (*N* = 129) and an overall smaller dose of cholecalciferol (equivalent of 7,143 IU/d) (125). In the intention to treat population (*N* = 129), vitamin D supplementation did not show a significant reduction in the ARR [rate ratio (rR) = 0.799, *p* = 0.38], which was the primary outcome measure. In patients who completed the study (*N* = 90), however, vitamin D supplementation led after 96 weeks to a significant reduction in the ARR (rR = 0.395, *p* = 0.01), in new T1 lesions (rR = 0.494, *p* = 0.03), and in disability progression as measured by the EDSS (−0.06 vs. 0.32, respectively, *p* = 0.03) in comparison to placebo (125). Since in both studies, SOLAR and CHOLINE, cholecalciferol was used as an add-on to interferon-beta, it is important to mention that in patients being treated with interferon-beta who received repeated MRI scans and measurements of serum 25(OH)D concentration, an inverse correlation between 25(OH)D and MRI activity was found before but not during treatment with interferon-beta (126). Other vitamin D supplementation studies investigating relapse risk, CNS lesions, and/or disability progression were either substantially smaller, or of shorter duration, or were not prospectively randomized and are therefore only listed in the reference section [see (127–130)]. Regarding the effects of vitamin D supplementation on fatigue and depression scores, Rolf et al. showed in a prospectively studied cohort of 40 MS patients that patients receiving cholecalciferol for 48 weeks did not improve significantly in fatigue or depression scores in comparison to placebo (131). In contrast, Achiron et al. found in a larger (*N* = 158), yet shorter (24 weeks of supplementation) prospective study a significant reduction of fatigue scores through the supplementation with alfacalcidol (132). Therefore, the effect of vitamin D supplementation on fatigue remains uncertain. To conclude, the vitamin D supplementation studies published so far were mostly insufficiently powered to detect significant differences in clinical disease parameters, especially since MS patients show a reduced serological and metabolic response to vitamin D supplementation and, therefore, may need higher doses to demonstrate clinically relevant effects (64, 133). Ongoing trials such as VIDAMS, PrevANZ, and D-Lay-MS, might shed new light on the efficacy of vitamin D supplementation in MS and CIS (134–137).

## Vitamin D Supplementation and MS Relapse Therapy

The immunological mechanism by which 1,25(OH)_2_D_3_ increases the effect of corticosteroids have led us to investigate whether 1,25(OH)_2_D_3_ increases the efficacy of methylprednisolone pulse therapy for the treatment of MS relapses (138). We demonstrated in human and murine CD3 + T cells that 1,25(OH)_2_D_3_ increases glucocorticoid receptor protein expression and consequently upregulates methylprednisolone induced apoptosis (138). *In vivo*, the combination therapy led to a significant decrease in active MOG_35–55_ EAE disease severity (138). Effects appear to be mediated via the glucocorticoid receptor because no difference was observed in animals specifically lacking glucocorticoid receptor expression in CD3 + T cells (138). Furthermore, in two different independent cohorts, patients with a steroid-resistant MS relapse had significantly lower 25(OH)D concentrations (138). The cellular pathway most plausible to explain this finding is the inhibition of mTOR because in mTORc1-deficient animals no synergistic effects were found, whereas treatment of wild type animals with mTOR inhibitors led to synergistic glucocorticoid effects. These findings may lead to a prospective clinical evaluation of 1,25(OH)_2_D_3_ in MS relapses because so far sufficient evidence for its use in this specific setting is lacking.

## Conclusion

Although certain parameters of radiological MS disease activity were significantly reduced by vitamin D supplementation as shown in randomized double-blind placebo-controlled trials, the evidence accumulated so far is not sufficient to allow drawing a definite conclusion on the effects of vitamin D supplementation on clinical parameters. The VIDAMS trial may provide further insights as it aims to investigate vitamin D supplementation in the yet largest (*N* = 172) prospectively randomized MS patient population followed up over ≥96 weeks (134). In addition, basic scientific research may increase our knowledge about the effects of vitamin D on the immune system. Considering the interactions between vitamin D and glucocorticosteroids, this knowledge may provide us with new therapeutic strategies for vitamin D administration in MS.

## Author Contributions

AM and RH contributed to the research of the literature and the writing and revision of the manuscript. MB and AC contributed to the writing and revision of the manuscript.

## Conflict of Interest

AC has received personal compensation for activities with Bayer, Biogen, Genzyme, Merck, Novartis, Roche, and Teva; received research support from the Swiss National Funds (SNF, No. 310030_172952), Genzyme, and UCB; and serves in the Editorial Board for Clinical and Translational Neuroscience and the Journal of International medical research. RH received research and travel grants from Novartis and Biogen Idec and received speaker’s honoraria from Biogen, Novartis, Merk, and Almirall. The remaining authors declare that the research was conducted in the absence of any commercial or financial relationships that could be construed as a potential conflict of interest.
